# Metagenomic analysis of the fecal microbiome in colorectal cancer patients compared to healthy controls as a function of age

**DOI:** 10.1002/cam4.5197

**Published:** 2022-09-03

**Authors:** Jordan Kharofa, Senu Apewokin, Theresa Alenghat, Nicholas J. Ollberding

**Affiliations:** ^1^ Department of Radiation Oncology University of Cincinnati Cancer Cancer Center Cincinnati Ohio USA; ^2^ Department of Infectious Disease University of Cincinnati College of Medicine Cincinnati Ohio USA; ^3^ Division of Immunobiology Cincinnati Children's Hospital Medical Center Cincinnati Ohio USA; ^4^ Department of Pediatrics University of Cincinnati College of Medicine Cincinnati Ohio USA; ^5^ Division of Biostatistics and Epidemiology Cincinnati Children's Hospital Medical Center Cincinnati Ohio USA

**Keywords:** Fusobacterium nucleatum, colibactin, young‐onset colorectal cancer

## Abstract

**Background and Aims:**

Colorectal cancer (CRC) incidence is increasing in young patients without a clear etiology. Emerging data have implicated the fecal microbiome in CRC carcinogenesis. However, its impact on young onset CRC is poorly defined.

**Methods:**

We performed a meta‐analysis of fecal metagenomics sequencing data from *n* = 692 patients with CRC and *n* = 602 healthy controls from eleven studies to evaluate features of the fecal metagenome associated with CRC. We hypothesized that known carcinogenic virulence factors (colibactin, fadA) and species abundance may be differentially enriched in young CRC patients relative to older CRC patients and controls.

**Results:**

Summary odds ratios (OR) for CRC were increased with the presence of colibactin (OR 1.92 95% CI 1.08–3.38), fadA (OR 4.57 95% CI 1.63–12.85), and *F. nucleatum* (OR 6.93 95% CI 3.01–15.96) in meta‐analysis models adjusted for age, gender, and body mass index. The OR for CRC for the presence of *E.coli* was 2.02 (0.92–4.45). An increase in the prevalence of *Fusobacterium nucleatum* (OR = 1.40 [1.18; 1.65]) and *Escherichia coli* (OR = 1.14 [1.02; 1.28]) per 10‐year increase in age was observed in models including samples from both CRC and healthy controls. Species relative abundance was differentially enriched in young CRC patients for five species‐*Intestinimonas butyriciproducens, Holdemania filiformis, Firimicutues bacterium CAG 83, Bilophilia wadsworthia, and Alistipes putredinis*.

**Conclusion:**

In this study, we observed strong associations with CRC status for colibactin, fadA, and *Fusobacterium nucleatum* with CRC relative to controls. In addition, we identified several microbial species differentially enriched in young colorectal cancer patients. Studies targeting the young CRC patients are warranted to elucidate underlying preclinical mechanisms.

## Graphical abstract

Differentially enriched bacterial species in young onset colorectal cancer patients.

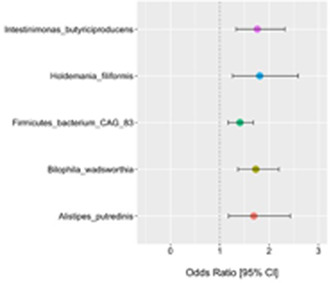

## 1 INTRODUCTION

Although the incidence and death rate from colorectal cancer has declined in patients older than 50 years of age with the adoption of screening, colorectal cancer incidence in young patients has been increasing in the United States^1^, ^2^ and world‐wide.^3^, ^4^, ^5^, ^6^, ^7^, ^8^ The etiology for the increased incidence in young patients remains unclear. Several studies evaluating the fecal metagenome have revealed enrichment in distinct microbial species in colorectal cancer patients when compared to healthy controls,^9^, ^10^, ^11^, ^12^ provoking the hypothesis that microbial features may, in part, explain the relative increase in young colorectal cancer incidence. Colorectal cancer develops within the presence of a diverse microbial environment. Emerging data has implicated the colorectal microbiome in mediating carcinogenesis through biofilm formation which may drive oncogenic pathways.^13^, ^14^, ^15^ Certain species harboring virulence factors have been shown to directly mediate carcinogenesis. These include colibactin producing *Escherichia coli* ^16^ and *Fusobacterium nucleatum*.^17^ Diet and potential interactions with the gastrointestinal microbiome have also been hypothesized as one possible etiology for increased young onset colorectal cancer incidence.^18^ Epidemiologic studies evaluating dietary patterns have observed higher rates of colorectal cancer in young and older patient populations with enriched sulfur microbial diets suggesting that metabolic byproducts from microbial sulfur metabolism may contribute to diet induced carcinogenesis.^18^, ^19^, ^20^ Despite the emerging understanding of how the fecal microbiome may influence colorectal carcinogenesis, its effect on the increasing incidence of young colorectal cancer patients is not well defined. In this study, we performed a meta‐analysis of publicly available fecal metagenomics sequencing data from 692 patients with colorectal cancer and 602 healthy controls to evaluate features of the colorectal cancer fecal metagenome. We hypothesized that the known carcinogenic virulence factors colibactin and FadA may vary with age in colorectal cancer patients relative to control populations and increase the risk of early onset CRC. In addition, we evaluate for unique species and microbial functional pathways enriched in young colorectal cancer patients relative to older patients and healthy controls.

## 2 MATERIALS AND METHODS

Metagenomic data were obtained using the curatedMetagenomicData package^21^ in R. The curatedMetagenomicData package provides uniformly processed human microbiome data from previously published works. The methods for generating the data provided with the package have been described previously in detail, but in brief, all raw sequencing data were downloaded after uniform taxonomic profiling via MetaPhlAn3^22^ and functional profiling via HUMAnN3^23^, ^24^ followed by manual curating of sample and study information. The work is exempt from IRB oversight as all data were de‐identified through publicly available sources. All included data were downloaded on November 18th, 2021 and reflect the data available in the curatedMetagenomicData snapshotDate(): 2021‐05‐18 files. Bacterial species relative abundance tables were obtained using the function curatedMetagenomicData::returnSamples(“relative_abundance”, counts = TRUE). MetaCyc pathway abundances were obtained using the function curatedMetagenomicData::returnSamples (“pathway_abundance”). UniRef90 gene families were obtained using the function curatedMetagenomicData::returnSamples(“gene_families”). Sample metadata and count matrices were converted to phyloseq objects prior to statistical analysis. All available studies were reviewed to identify samples containing colorectal cancer patients and healthy controls. Eleven datasets were identified meeting these criteria.^9^, ^10^, ^11^, ^12^, ^18^, ^25^, ^26^, ^27^, ^28^, ^29^ In all studies, stool samples were obtained prior to therapy in included cases of colorectal cancer or adenoma and controls. Patients with invasive cancer or adenoma are classified as colorectal cancer (CRC) within the curatedMetagenomicData package.

### 2.1 Statistical analysis

Participant and study‐level characteristics were described using means and standard deviations (SD) for continuous variables and frequencies and percents for categorical variables. The prevalence of sequence reads mapping to the UniRef90 fadA gene family (UniRef90_Q5I6B0), any colibactin gene family (Table 1), *F.nucleatum*, and *E.coli* were examined according to colorectal cancer status and age in 10 year bins using stacked bar charts. Generalized linear mixed effects regression (GLMER) was used to model the predicted prevalence of gene families and taxa as a non‐linear function of age. Estimates were obtained using GLMER with logit link function to model the binomially distributed responses; a study‐specific random intercept; and fixed‐effect terms for age, cancer status (CRC vs. healthy control), and the age by cancer status interaction. Age was modeled using restricted cubic spline terms with four degrees of freedom (d.f.) with default knot placement using the splines package (version 4.1.1). Models were fit using the glmmTMB (version 1.1.2) package in R and conditional effect plots generated using the sjPlot (version 2.8.9) package to visualize the prevalence as function of age and cancer status. A two‐stage individual patient data meta‐analysis (IPDMA) approach was used to estimate summary odds ratios (OR) and 95% confidence intervals (CI) for colorectal cancer case status according to the presence of fadA and colibactin gene families, *F.nucleatum*, and *E.coli*. Logistic regression was used to obtain the log odds and standard errors (SE) for each study separately. Model terms included the exposure of interest, age, gender, and body mass index (BMI). Age and BMI were modeled using restricted cubic spline terms with 3 d.f. Summary ORs were obtained using the metafor (version 3.0.2) package in R and estimated via restricted maximum likelihood (REML) with the Hartung‐Knapp estimate of variance. Two‐stage IPDMA with adjustment for gender, CRC status, and body mass index was also used to specifically obtain conditional summary ORs for the linear increase in prevalence per 10‐year increase in age at the time of sample collection. Interaction ORs assessing the difference in the effect of each gene or taxa on CRC case status according to age < 50 years at onset (i.e., young onset) or older were obtained by pooling the log odds and SE for the cross‐product terms for age (binary) and the exposure of interest in the second stage meta‐analysis. Studies contributing zero weight to the summary OR were removed from all forest plots for clarity. Due to the small number of studies including participants <50 years of age, summary interaction ORs could not be estimated for fadA, colibactin, or *F.nucleatum*. Logistic regression was used to estimate and visualize the potential non‐linear association for colibactin and *F.nucleatum* for the data in Yachida 2019 alone. GLMER as described above was used to estimate the pooled association for interaction between continuous age and *E.coli*. Given the small number of studies, and exposure/outcome combinations, one‐stage meta‐analysis estimates were also conducted as a sensitivity analysis. Main effects models were fit using GLMER and were estimated allowing for stratified intercepts, study‐specific centering, a random‐effect term for the exposure of interest, and uncorrelated random‐effects. Summary estimates of the within study interaction were obtained similarly, but by partitioning the within‐ and across‐study contributions via study‐specific centering as described by Riley et al.^30^

**TABLE 1:** Participant characteristics

	Control (*n* = 609)	CRC (*n* = 692)
Age (years), mean (SD)	59.68 (12.49)	63.25 (10.97)
Age < 50 years, *n* (%)	120 (19.7)	81 (11.7)
Male, *n* (%)	329 (54.0)	438 (63.3)
Body mass index, mean (SD)	23.72 (3.53)	24.33 (4.30)
Study, *n* (%)		
Zeller G (2014)	59 (9.7)	51 (7.4)
Feng Q (2015)	16 (2.6)	46 (6.6)
Yu J (2015)	37 (6.1)	74 (10.7)
Vogtmann E (2016)	52 (8.5)	49 (7.1)
Hannigan GD (2017)	27 (4.4)	25 (3.6)
Thomas AM (2018a)	12 (2.0)	28 (4.0)
Thomas AM (2018b)	27 (4.4)	31 (4.5)
Wirbel J (2018)	65 (10.7)	60 (8.7)
Gupta A (2019)	30 (4.9)	30 (4.3)
Thomas AM (2019)	40 (6.6)	40 (5.8)
Yachida S (2019)	244 (40.1)	258 (37.3)
Country, *n* (%)		
AUT	16 (2.6)	46 (6.6)
CAN	3 (0.5)	2 (0.3)
CHN	37 (6.1)	74 (10.7)
DEU	65 (10.7)	60 (8.7)
FRA	59 (9.7)	51 (7.4)
IND	30 (4.9)	30 (4.3)
ITA	39 (6.4)	59 (8.5)
JPN	284 (46.6)	298 (43.1)
USA	76 (12.5)	72 (10.4)
Presence fadA or colibactin, *n* (%)	70 (11.5)	148 (21.4)
UniRef90_Q5I6B0: Adhesion A (fadA), *n* (%)	5 (0.8)	45 (6.5)
Presence colibactin, *n* (%)	65 (10.7)	116 (16.8)
Presence *Fusobacterium nucleatum*, *n* (%)	16 (2.6)	155 (22.4)
Presence *Escherichia coli*, *n* (%)	399 (65.5)	522 (75.4)

Shannon diversity was calculated from the species abundance data in phyloseq after subsampling to the lowest observed read depth (2,291,325 reads). Two‐stage IPDMA were conducted to obtain main effect and interaction (e.g., young onset) ORs for CRC status according to a 1 SD increase in Shannon diversity. Principal coordinates ordination performed on the between sample Bray–Curtis dissimilarity matrix was performed to assess clustering of samples based on CRC status and age. Two‐stage IPDMA were also used to obtain summary and interaction ORs for a 1 SD increase in the relative abundance of each species and MetaCyc pathway detected in at least 30% of participant samples. MetaCyc pathways abundances were truncated at the 97th percentile to limit the impact of outlying observations on model estimates. Library size normalization for the species abundance data was performed using the geometric means of pairwise ratios approach (GMPR)^31^ and R package (version 0.1.3) developed for microbiome data to account for differences in read depth and truncation of normalized counts at the 97th percentile prior to modeling. All analyses were conducted using R version 4.1.1.

## 3 RESULTS

**FIGURE 1:**
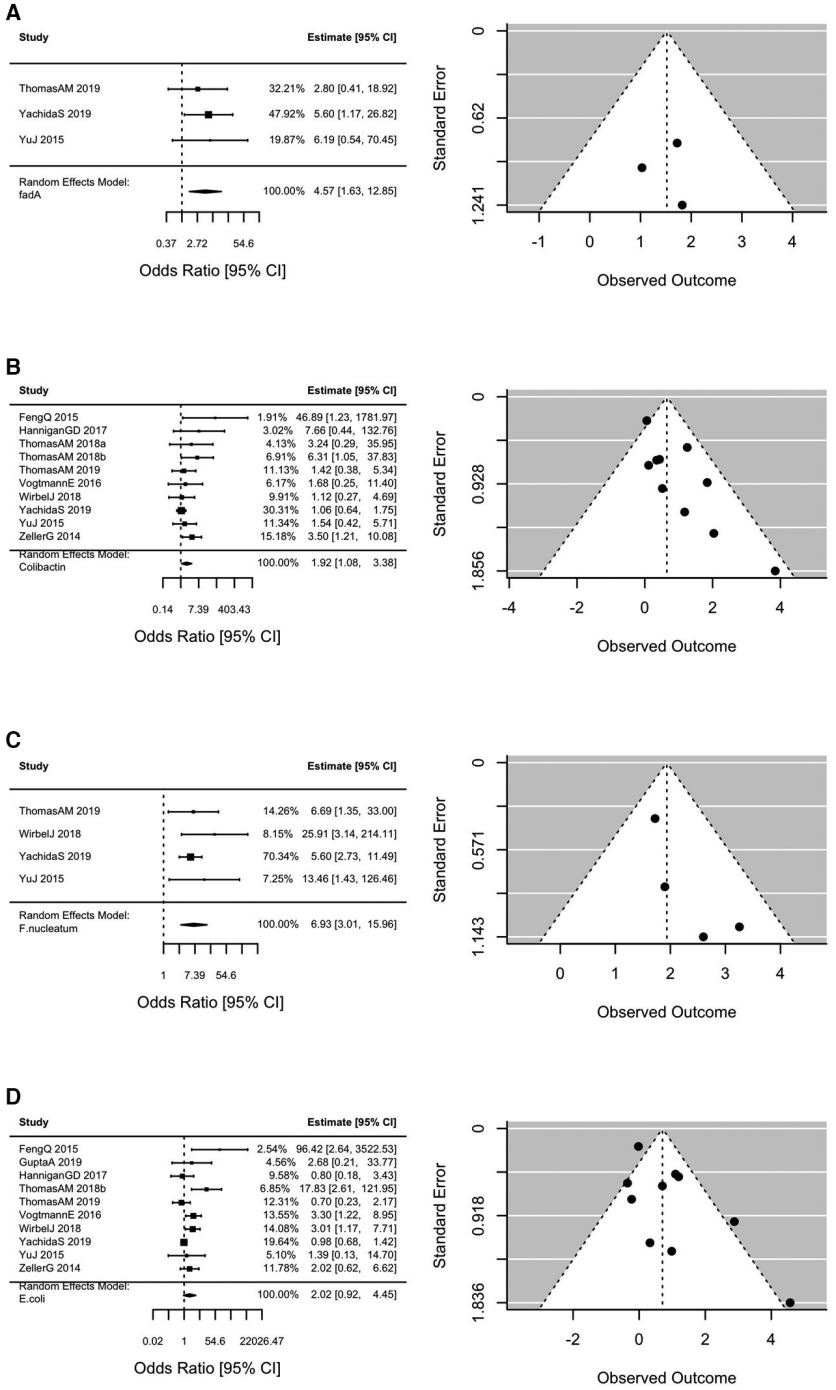
Odds ratios (OR) and 95% confidence intervals (CI) for colorectal cancer status according to the presence of fadA gene families, colibactin gene families, *F. nucleatum*, and *E. coli*. Study estimates providing zero weight to the summary OR not shown. Funnel plots present the log odds plotted against the log standard error. (A) FadA. (B) Colibactin. (C) *F. nucleatum*. (D) *E. coli*.

The study demographics, patient characteristics, and presence of virulence factors in CRC patients and controls are summarized in Table 1. A total of 692 patients with CRC and 609 controls were included in the analysis across 11 studies with patients represented from nine countries. The summary odds ratios (OR) and 95% confidence intervals (CI) for colorectal cancer status according to the presence of fadA gene families, colibactin gene families, *F. nucleatum*, and *E. coli* are displayed in Figure 1. The odds of CRC were increased with the presence of colibactin (OR 1.92 95% CI 1.08–3.38), fadA (OR 4.57 95% CI 1.63–12.85), and *F. nucleatum* (OR 6.93 95% CI 3.01–15.96). The summary OR for CRC for the presence of *E.coli* was 2.02 (95% CI 0.92–4.45). Similar results were obtained from the one‐stage models.

**FIGURE 2:**
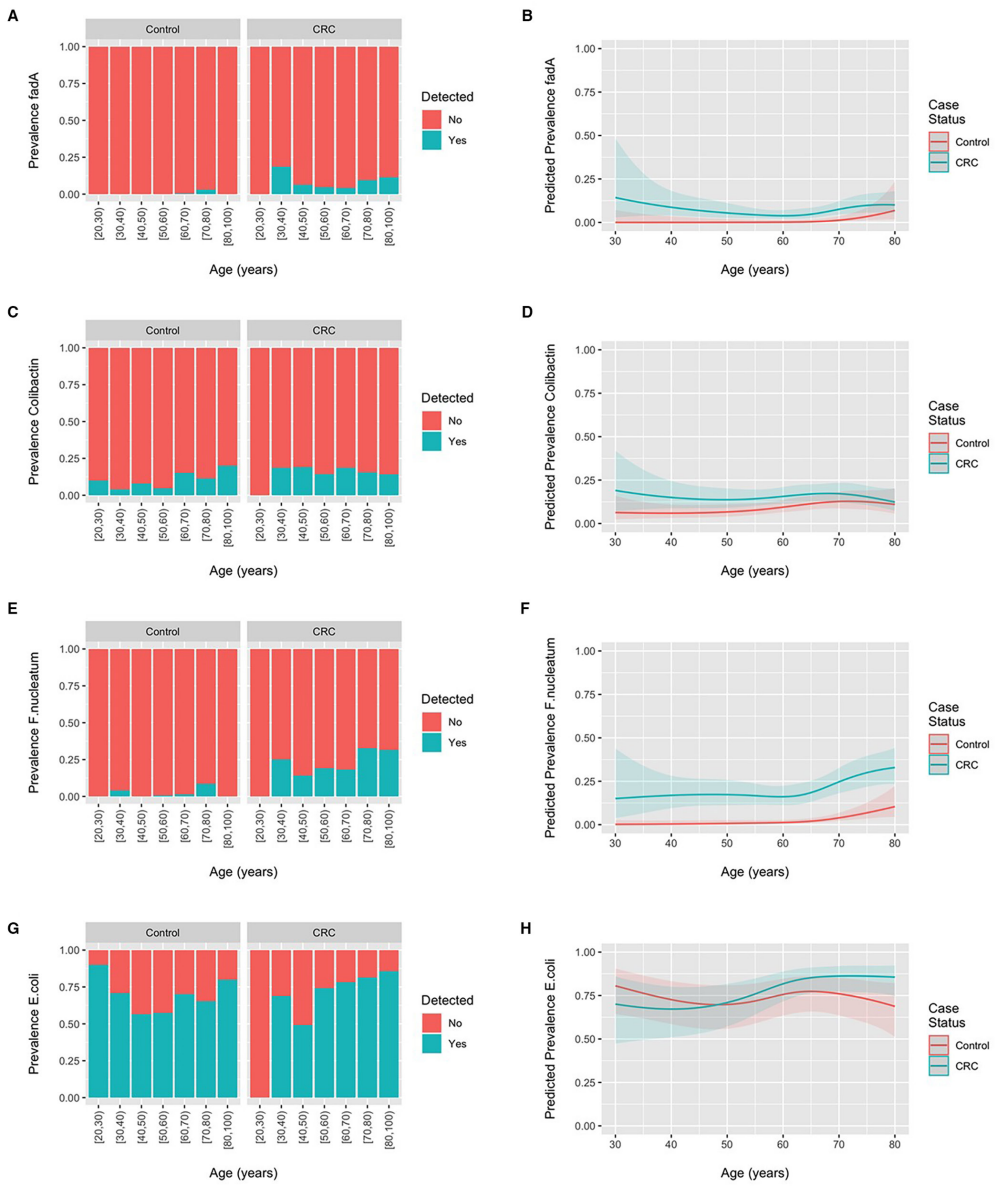
Prevalence of fadA gene families, colibactin gene families, *F. nucleatum*, and *E. coli* across all studies according to age and colorectal cancer status. (A, C, E, F) Observed prevalence according to participant age in 10‐year age bins. (B, D, F, H) Predicted prevalence estimated using generalized linear mixed‐effects regression. Shaded bands reflect 95% confidence intervals.

**FIGURE 3:**
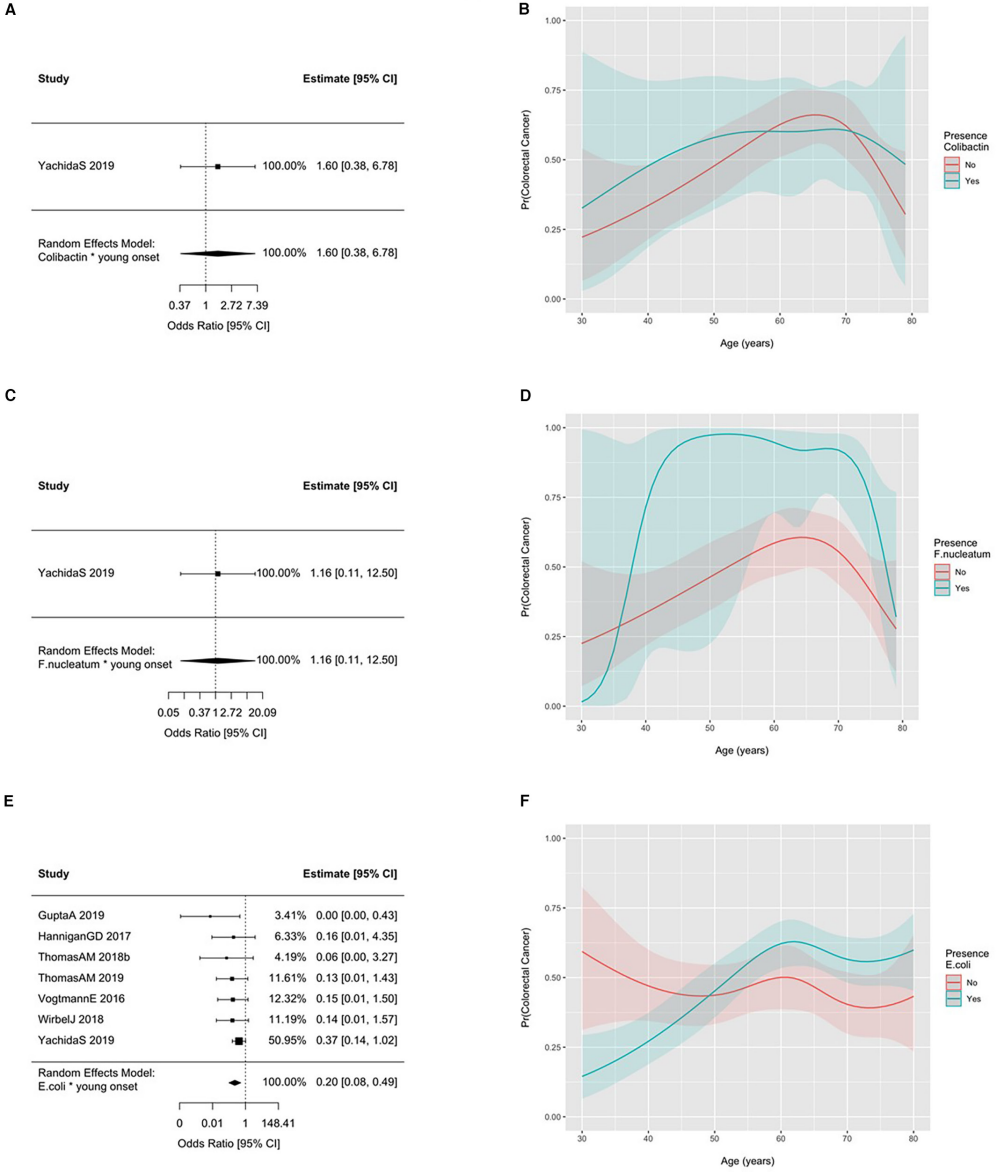
Odds ratios (OR) and 95% confidence intervals (CI) for colorectal cancer status according to the presence of colibactin gene families, *F. nucleatum*, and *E. coli* and age less than 50 years (i.e., interaction odds ratios for young onset disease). Study estimates providing zero weight to the summary OR not shown. Summary interaction odds ratios could not be obtained for fadA, colibactin gene families, or *F. nucleatum*. Predicted probability of colorectal cancer status according to presence of colibactin gene families, *F. nucleatum*, *E. coli* and age as estimated from generalized linear mixed‐effects regression. Gray bands reflect 95% confidence intervals. (A) Colibactin. (B) *F. nucleatum*. (C) *E. coli*.

The prevalence of fadA gene families, colibactin gene families, *F. nucleatum*, and *E. coli* across all studies according to age and colorectal cancer status are displayed in Figure 2. No material difference in the prevalence of these gene families or taxa between CRC or healthy controls was observed across the age spectrum. However, the odds of detecting *F. nucleatum* (OR = 1.40 [1.18; 1.65] per 10‐year increase in age) and *E. coli* (OR = 1.14 [1.02; 1.28]) were increased when age was modeled as a linear term in models including samples from both CRC patients and healthy controls. ORs were 1.26 (0.96; 1.67) for fadA gene families and 1.08 (0.93; 1.25) for colibactin gene families for a 10‐year increase in age. Figure 3 displays the summary interaction ORs and predicted probabilities of colorectal cancer status for young, when compared to older participants, according to presence of *E. coli* and age as estimated from the two‐stage meta‐analysis and generalized linear mixed‐effects regression. The presence of *E. coli* was associated with lower probability of CRC in younger patients (OR 0.20 95%CI 0.08–0.49) when compared to older patients. No pooled estimates could be obtained colibactin gene families and *F. nucleatum* due to the low number of participants with CRC and the presence of these gene families and taxa. The ORs for the individual studies in which these associations could be estimated with sufficient precision are presented in Figure 3 and provide an interaction OR of 1.60 (0.38; 6.78) for colibactin genes and 1.16 (0.11; 12.50) for *F. nucleatum*. Consistent results were obtained for the one‐stage models.

**FIGURE 4:**
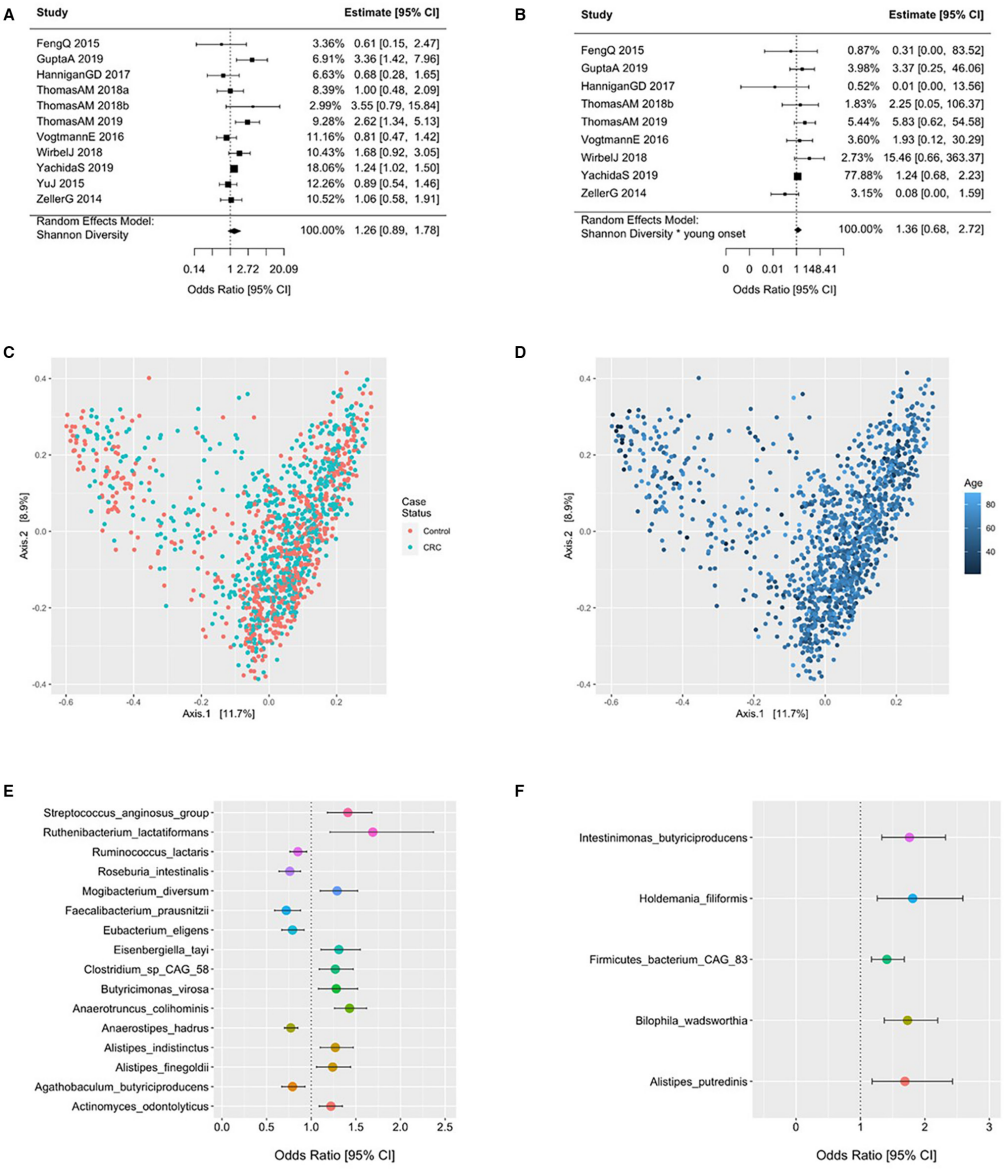
Associations with colorectal cancer (CRC) according to bacterial species diversity and differential abundance. (A) Odds ratios (OR) and 95% confidence intervals (CI) for colorectal cancer status according to a one standard deviation (SD) increase in Shannon diversity. (B) Interaction OR and 95% CI for CRC according to a one SD increase in Shannon Diversity for those 20–49 years of age (early onset) when compared to those 50 years of age or older. (C, D) Principal coordinates ordination performed on the species‐level Bray–Curtis dissimilarity. (E) OR and 95% CI for CRC status according to a one SD increase in species relative abundance. Species with an FDR corrected *p*‐value <0.1 shown. (F) Interaction OR and 95% CI for CRC according to a one SD increase in species relative abundance for those 20–49 years of age (early onset) when compared to those 50 years of age or older. Species with an FDR corrected *p*‐value <= 0.25 shown.

**FIGURE 5:**
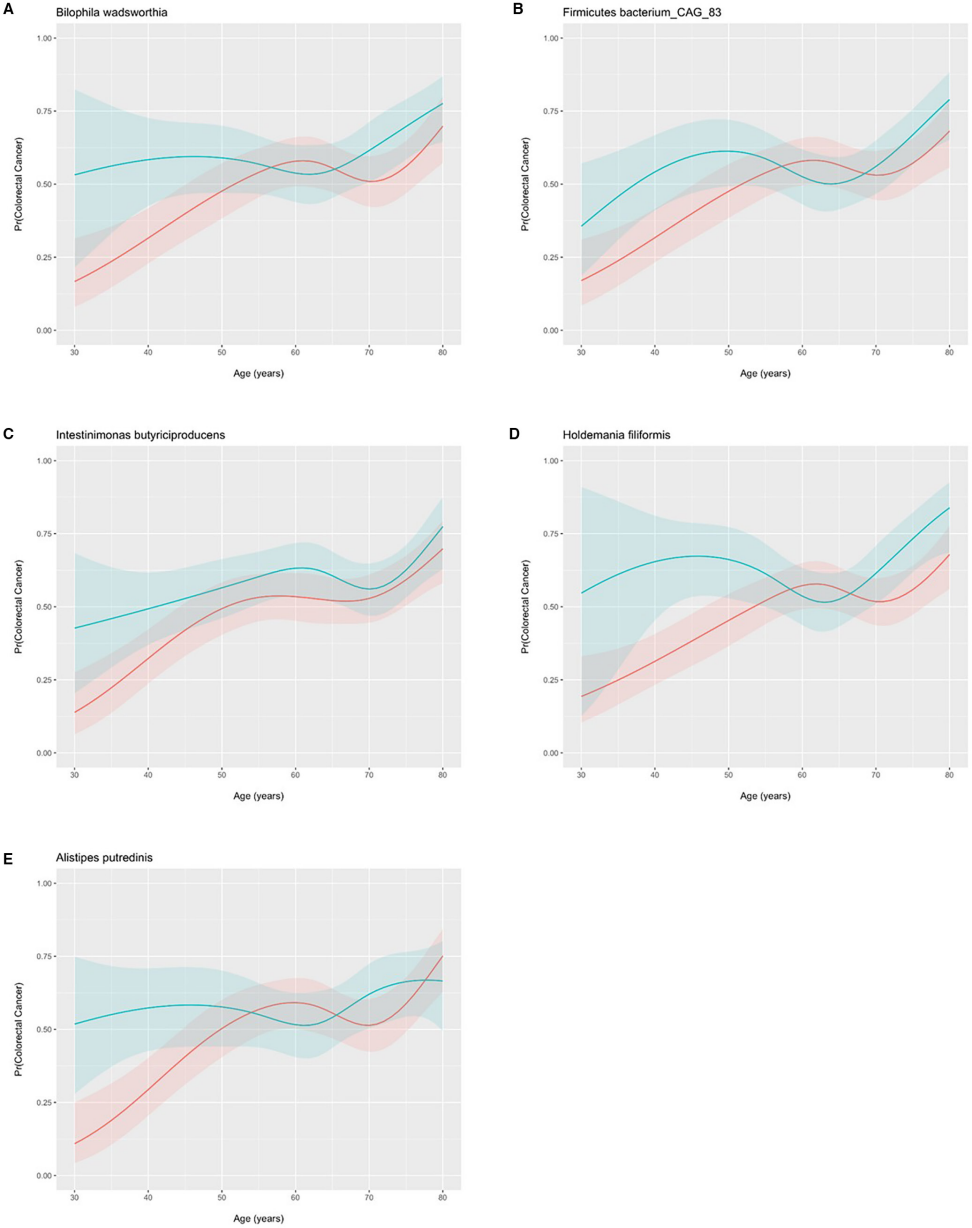
Predicted probabilities for colorectal cancer status according to species relative abundance and age obtained from generalized linear mixed‐effects regression. Shaded bands reflect 95% confidence intervals (CI).

**FIGURE 6:**
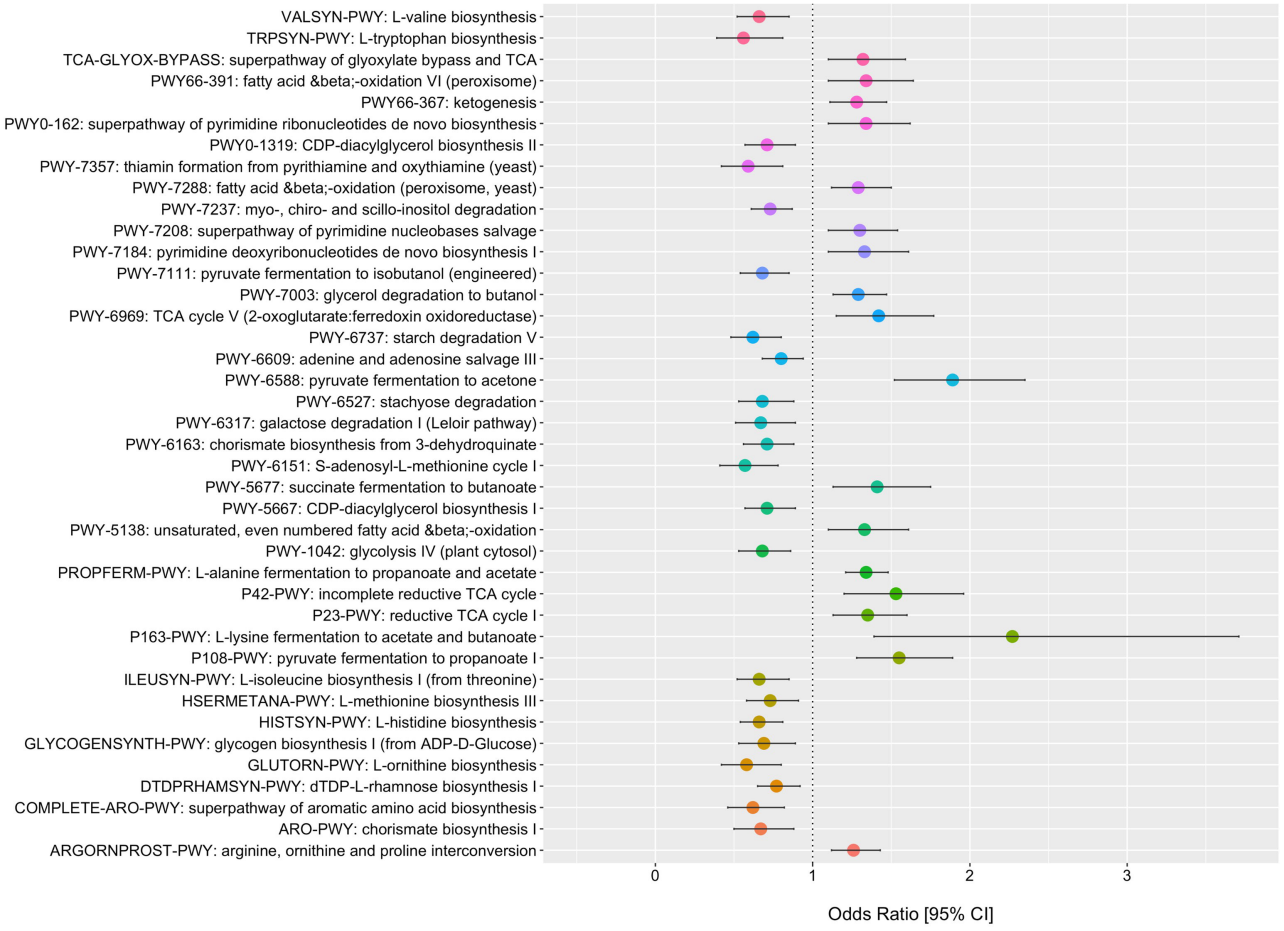
OR and 95% CI for CRC status according to a one SD increase in MetaCyc pathway relative abundance. Pathways where FDR *p*‐value <0.10 shown.

Associations with colorectal cancer (CRC) according to bacterial species diversity are examined in Figure 4 **(**A‐D). The Shannon diversity index did not influence the odds of CRC for all patients (Figure 4A) with no interaction in patients <50 years old (Figure 4B). The principal coordinates ordination based on the Bray–Curtis dissimilarity for Shannon diversity are displayed in Figure 4C for CRC and controls and Figure 4D as a function of age with no material clustering observed by age or disease status. Species differences in CRC patients relative to controls are shown in 4E and for species by age of onset interaction in Figure 4F and in supplementary Tables S1 and 2. Interaction ORs for CRC onset prior to age 50 according to a one standard deviation (SD) increase in species relative abundance are shown for those species with a FDR‐corrected *p*‐value <= 0.25. Species enriched in CRC patients 20–49 years of age include *Intestinimonas butyriciproducens, Holdemania filiformis, Firimicutues bacterium CAG 83, Bilophilia wadsworthia, and Alistipes putredinis*. For enriched species in young CRC patients, Figure 5 displays predicted probabilities for CRC according to species relative abundance and age obtained from generalized linear mixed‐effects regression. OR and 95% CI for CRC status for MetaCyc metabolic pathways are shown in Figure 6 for pathways with an FDR *p*‐value <0.1 and in supplemental Table S3. Interaction ORs did not provide support (FDR *p*‐values >0.74) that associations between CRC status and MetaCyc pathways differed for younger (< 50 years of age) when compared to older participants (Table S4).

## 4 DISCUSSION

Recently, screening guidelines have changed from 50 years to 45 years.^32^ However, the colorectal cancer incidence in the United States and other western populations in patients younger than age 45 years continues to increase.^1^, ^33^ Using the Surveillance, Epidemiology, and End Results (SEER) database, investigators noted a 1%–3.2% annual increase in colorectal cancer in patients aged 20–39 years since the mid‐1980s.^1^ The etiology for the increasing rates of young colorectal cancer patients remains unclear. Although young patients are more likely to have hereditary pathogenic mutations, no genetic etiology is observed in 65% of patients <35 years of age and 80% of patients less than 50 years of age.^2^ Additionally, genetic etiologies do not explain the relative increase over time in young patient populations. In this analysis, we evaluate variation in the fecal metagenome across the colorectal cancer age spectrum as well as variations in known microbial‐derived virulence factors (colibactin and FadA) which have been previously associated with carcinogenesis.

Colibactin has previously been implicated in colorectal carinogenesis through DNA alkylation and double strand break formation. In an organoid model of colorectal cells, injection of colibactin producing (PKS+) Escherichia Coli induced mutational signatures which were similar to signatures noted in clinical specimens suggesting a causative role in carcinogensis.^16^ *Fusobacterium nucleatum* and its virulence factor fadA has also been observed to mediate carcinogenesis in preclinical models.^17^ Our results confirm these preclinical findings with higher prevalence of colibactin (OR 1.92 95% CI 1.08–3.38), fadA (OR 4.57 95% CI 1.63–12.85), and *Fusobacterium nucleatum* (OR 6.93 95% CI 3.01–15.96) in CRC patients relative to control patients. Due to limited number of younger patients across the individual studies, our statistical power was limited in estimating differences in these associations according to age of onset for these gene families and taxa using generalized linear mixed effect regression models. However, when age was modeled as a linear term there was a modest increase in *Fusobacterium nucleatum* (OR = 1.40 [1.18; 1.65]) and *Escherichia coli* (OR = 1.14 [1.02; 1.28]) per 10‐year increase in age for CRC and control participants combined. These results are congruent with a Chinese cohort of colorectal cancer patients.^34^ Using lower resolution 16S sequencing, investigators noted enrichment of *Fusobacterium nucleatum* in older patients>50 years of age rather than the younger cohort.

In addition to known virulence factors, we examined species enriched in young CRC patients (20–49). Strengths of our approach include uniformly processed metagenomic data from multiple international cohorts. Using metagenomic data, more granular species resolution can be obtained compared to 16S sequencing. Additionally, as the microbiome is known to change with age it is critical to account for these effects when evaluating unique species differences in young CRC patients relative to older CRC populations. In this meta‐analysis, healthy control populations across the age spectrum allowed evaluation of species differentially enriched in young CRC patients relative to older CRC patients and controls. Five species were identified as being differentially enriched in patients 20–49 years of age including *Intestinimonas butyriciproducens, Holdemania filiformis, Firimicutues bacterium CAG 83, Bilophilia wadsworthia, and Alistipes putredinis*. Additional work is needed to fully understand the implication of these species enriched in young CRC patients.

Dietary factors have been postulated as a potential mediator increased young CRC increase. Potentially supporting this hypothesis, epidemiologic data within the United States have revealed that magnitude of increase in CRC incidence varies by region with higher rates observed in the Southeast congruent with higher obesity prevalence.^2^ Although young onset CRC are often not obese, these differences may potentially implicate dietary factors. The finding of enriched *Bilophilia wadsworthia* in young colorectal cancer patients in our analysis is intriguing given its role in sulfur metabolism and production of hydrogen sulfide. Hydrogen sulfide has been observed to have carcinogenic potential by directly damaging DNA in mammalian cells or indirectly by disrupting the normal colonic mucosal layer.^35^, ^36^, ^37^, ^38^

Adherence to a sulfur microbial diet rich in processed meats, but low in vegetables and legumes, has been associated with an increased risk of colorectal cancer in multiple epidemiologic cohorts.^18^, ^19^, ^20^ In a prospective cohort of 307 men from the Men's Lifestyle Evaluation Study, investigators prospectively measured dietary patterns as well as fecal metagenomics and metatranscriptomes to evaluate species enriched when patients adhere to a sulfur microbial diet.^19^ *Bilophilia wadsworthia* was one of two notable species enriched in patients with a sulfur microbial diet possessing unique sulfur fixation enzymes such as hydrogen sulfite reductase. In a separate study of 30,818 women less than 50 years of age, investigators examined dietary patterns and association with early onset CRC. They noted that higher sulfur microbial diet scores in early adulthood were associated with increased risk of early‐onset conventional adenomas.^18^ Stool samples in this cohort were unavailable. Although the dietary patterns of patients in our study are unknown, enrichment of *Bilophilia wadsworthia* in young CRC patients is consistent with these findings. Further work is needed to establish causal mechanism and examine potential confounders.

We also observed an enrichment of *Alistipes putredinis* and *Intestinimonas butyriciproducens* in young CRC patients in this analysis. *Alistipes* is classified as anaerobic, gram‐negative genus first described in 2003 isolated from children with appendicitis.^39^ It has been implicated in promoting colonic inflammation and carcinogensis in preclinical models.^40^, ^41^ Lcn2 is an antimicrobial factor expressed in colonic cells. In Lcn2 knockout models, Moschen et al observed that *Alistipes putredinis* could potently induce inflammation and tumorigenesis potentially through the IL‐6‐STAT3 pathway.^41^ *Intestinimonas butyriciproducens* is a prevalent butyrate producing species in the human gut.^42^ The role of butyrate in colorectal carcinogenesis remains controversial with models suggesting tumorigenesis and anti‐inflammatory effects.^43^ Thus, there is preclinical evidence which provide biologic plausibility implicating these enriched species in mediation of colorectal carcinogenesis in young patient populations. Additional work is needed to explore causal mechanisms and to better understand host‐diet‐microbial interactions.

Our analysis has several limitations. The curatedMetagenomicData sample data do not include information on tumor genomic details or sidedness. Thus, we are unable to examine left‐ versus right‐sided lesions and the potential for species‐specific interactions in carcinogenesis related to anatomic location. Additionally, both adenomas and invasive colorectal cancers are coded as CRC within the dataset so we are unable to assess unique differences that may exist along the spectrum of colorectal cancer carcinogenesis. This study represents a cross sectional assessment of species prevalence at the time of study enrollment. Therefore, we are unable to account for transient species that may have been present earlier in life. In addition, the statistical power to detect whether associations between CRC status and metagenomic features differed between younger and older participants was low as evidenced by the small number of individual studies in which several associations could even be estimated. Thus, future work specifically targeting younger patients is required to better understand the role of metagenomic factors in the etiology of early onset CRC. Lastly, when pooling estimates assuming a random intercept and shared effects of predictors across studies to visualize potential non‐linear associations between CRC patients and healthy control both the across‐ and within‐trial information contributes to the pooled interaction term.

## 5 CONCLUSION

In this analysis, we performed a meta‐analysis of high‐resolution, fecal metagenomics sequencing data from 692 patients with colorectal cancer and 609 healthy controls to evaluate features of the colorectal cancer fecal metagenome across the age spectrum. Our results confirm strong association of colibactin, fadA, and *Fusobacterium nucleatum* presence in the fecal metagenome in CRC patients relative to controls. We found a modest increase in *Fusobacterium nucleatum* and *Escherichia coli* with increasing age; however, we had limited power to accurately estimate pooled ORs in young CRC patients. However, species relative abundance was differentially enriched in young CRC relative to older CRC patients and healthy controls for five species (*Intestinimonas butyriciproducens, Holdemania filiformis, Firimicutues bacterium CAG 83, Bilophilia wadsworthia, and Alistipes putredinis)* with several potential mechanisms supported by preclinical data. Given the alarming increase in young onset of colorectal cancer without clear etiologies, additional studies specifically targeting the young colorectal cancer patient population are warranted to examine these findings.

## AUTHOR CONTRIBUTIONS

Kharofa: Concept, Data interpretation, Manuscript drafting.

Apewokin: Data interpretation, Manuscript drafting.

Alenghat: Data interpretation, Manuscript drafting.

Ollberding: Concept, Analysis, Data interpretation, Manuscript drafting.

## FUNDING INFORMATION

None.

## CONFLICT OF INTEREST

Kharofa: None.

Apewokin: None.

Alenghat: None.

Ollberding: None.

## Supporting information


Table S1

Table S2

Table S3

Table S4

## Data Availability

All data used for this manuscript are available through the curatedMetagenomicData package (version 3.0.10) in the R environment for statistical computing and graphics.
